# Analytical Ultracentrifugation in Aotearoa: A Brief History of the Technique and its Place Among Other Tools Used to Investigate Biomolecular Interactions

**DOI:** 10.1002/snz2.70063

**Published:** 2026-07-09

**Authors:** Liam S. Turk, Zachary D. Tillett, Rebecca E. McKenzie, Sarah L. Draper, Gavin F. Painter, Renwick C. J. Dobson

**Affiliations:** ^1^ Biomolecular Interaction Centre, the MacDiarmid Institute and School of Biological Sciences University of Canterbury | Te Whare Wānanga o Waitaha Christchurch New Zealand; ^2^ Malaghan Institute of Medical Research Wellington New Zealand; ^3^ Ferrier Research Institute Victoria University of Wellington Lower Hutt New Zealand; ^4^ Department of Biochemistry and Pharmacology Bio21 Molecular Science and Biotechnology Institute University of Melbourne Parkville Victoria Australia

**Keywords:** analytical ultracentrifugation (AUC), biomolecular interactions, DNA, proteins

## Abstract

Analytical ultracentrifugation (AUC) is a powerful and versatile tool for studying biomolecular interactions. The purpose of this commentary is to provide foundational knowledge of the technique and its uses through the presentation of two case studies in which AUC provides key data to answer research questions. The first case study concerns a practical formulation problem associated with mRNA‐based vaccines. The second case study showcases how we tackle the problem of defining the properties of a protein–DNA interaction that regulates gene expression. We place AUC in the context of other biophysical techniques that we use in the Biomolecular Interaction Centre, based at the University of Canterbury in Ōtautahi Christchurch. We hope this work may prove helpful for protein biochemists and biophysicists in Aotearoa New Zealand in selecting appropriate approaches for investigating biomolecular interactions and encourage them to contact us if any of the resources presented herein may be useful to them. We also use a portion of this commentary to acknowledge and document the foundational contributions of the early innovators in AUC in Aotearoa New Zealand.

## 1 Introduction

Biology is in part a product of molecular interactions. Biomolecules (e.g., proteins, DNA, RNA, lipids, and carbohydrates) come together and form the complex cellular machinery that collectively becomes the organism. In the adjective *biomolecular*, *bio‐* refers to life, ‐*molecular* refers to a scale. A biomolecular interaction is the coming together of very small, submicroscopic, units that are linked to life. Some of these units, usually referred to as particles, exist in the everyday vernacular.

Take for example, DNA. At its most basic, it is the genetic code that makes us individuals. However, we may not appreciate that our general understanding of DNA hinges on biomolecular interactions. Genomic DNA, which encodes our genes, comprises very large structures where the individual units (i.e., nucleotides) are connected to each other by phosphodiester bonds. The highly recognisable double helix structure forms as two strands of complementary DNA sequences interact such that they are stabilised by nucleotide base pairing. The genetic information that is contained within the specific nucleotide sequence is thus buried within the double helix and protected from the environment by the phosphate backbone. Furthermore, DNA exists in different shapes or conformations, and B‐DNA is the most common form (Watson and Crick 1953; Dickerson et al. 1982; Wang et al. 1979). Characteristic of B‐DNA is the alternating presence of major and minor grooves within its structure. These grooves can be thought of as a molecular topology and serve as landing points for proteins that might alter gene expression. The DNA double helix is an elegant example of layered biomolecular interactions forming the fundamental building blocks of life.

From an applied perspective, knowledge of biomolecular interactions serves as the basis for many technologies. The development of monoclonal antibodies to treat human diseases like cancers, autoimmune disorders, or immune‐mediated diseases is an obvious example. These drugs form a direct antibody–target interaction, altering the biology of the target to provide a therapeutic benefit. A well‐established monoclonal antibody target is the programmed cell death protein 1/programmed cell death ligand 1 (PD‐1/PD‐L1) interaction, which is an immune checkpoint. Under physiological conditions, this interaction serves to hinder the immune system from attacking one's own cells. However, in certain cancers it is therapeutically beneficial to stop this interaction because cancer cells may also harness this checkpoint. An antibody that tightly binds to either PD1 or PD‐L1 and blocks the interaction promotes the immune system's response against cancerous cells that may otherwise have evaded the immune system (Lee 2024).

Humans have also employed other technologies well before it was understood that biomolecular interactions were the underlying forces at work. For example, cheesemaking has long relied on an enzymatic reaction to curdle milk in its process. Rennet is an extract derived from the stomachs of ruminants and contains a mixture of enzymes, among them chymosin, that cleave the protein casein into an insoluble form. To do this, chymosin must bind to and interact with the casein proteins. Now, most cheese is produced using recombinant chymosin made by microbes, but certain French cheeses are required to use traditional rennet (Décret du 30 décembre 1998).

These are very limited examples, but we hope the reader appreciates how understanding biomolecular interactions serves to help us as a society. Within the Biomolecular Interaction Centre at the University of Canterbury | Te Whare Wānanga o Waitaha, we specialise in the measurement and analysis of biomolecular interactions and are fascinated by the plethora of examples that drive living systems.

Measuring events on the molecular scale has, unsurprisingly, many challenges. One of the more obvious challenges is that the objects we are trying to analyse are small. How small are they? A grain of sand (diameter ∼0.5 mm or 500,000 nm) is about 100,000 times larger than the average protein (diameter ∼50 Å or 5 nm). In relatable terms, if we scale the grain of sand up to the size of Ōtautahi Christchurch (∼20 km in diameter), then a protein would be approximately the size of a football. Of course, not all biomolecular interactions that we measure occur between molecules that are the size of the average protein, some are smaller (e.g., peptides and small molecules), and some are larger (e.g., lipid nanoparticles (LNPs) and viruses). Differences in size can further complicate the way we measure and analyse data, for example if the observed signal increases in a nonlinear relationship to size (as is the case when using light scattering as a measurement tool) and require mathematical manipulation to understand.

Two other challenges that scientists have had to work through are that many of these interactions occur quickly and that they can occur between molecules of differing physicochemical properties. It is one thing to measure the interaction between an antibody and the specific protein antigen it binds; this sort of interaction is typically stable and occurs between two proteins. But what if the interaction is weak and occurs between two distinct biomolecules, such as DNA and lipids? Or what if there are three interacting molecules?

The purpose of this review is, in part, to showcase recent and ongoing case studies that might help emerging researchers navigate the options available when deciding how to study biomolecular interactions. We provide foundational information on analytical ultracentrifugation (AUC), a technique that in our opinion is particularly useful. Within these case studies, we highlight the resources available at the Biomolecular Interaction Centre and encourage scientists in Aotearoa New Zealand to contact us if these methods may prove useful for their own research goals.

## 2 Main Text

### 2.1 Biomolecular Interactions

#### 2.1.1 The Molecular Forces that Mediate Biomolecular Interactions

Biological environments such as the cytoplasm or blood behave like colloidal systems, where proteins and other macromolecules are dispersed within a solution. At high concentrations, the spatial proximity of these molecules decreases (i.e., they are closer to one another), and electrostatic interactions become critical for how these molecules interact. These forces extend beyond the *van der Waals surface* and include charge–charge, charge–dipole, and dipole–dipole interactions. Among these, charge–charge interactions are the longest‐range and can exert significant influence even at nanometre distances, shaping molecular organisation and stability in crowded cellular conditions.

*Van* *der Waals forces* are distance‐dependent attractive or repulsive forces. Shifts in molecules’ electron distributions create transient dipoles that are attractive at moderate distances. As molecules approach the *van der Waals distance*, the force becomes repulsive due to the overlap of electron clouds. A molecule's *van der Waals surface* is a representation of the distance to which another molecule might approach before repulsive forces limit any further interaction.

Proximity energies arise from multiple forces acting between adjacent biomolecules. While the *van der Waals interaction* at close proximity is always repulsive, most other forces—such as dipolar and hydrophobic interactions—tend to be attractive. Charge–charge interactions can be either repulsive or attractive depending on the sign of the charges involved. This balance of forces determines whether molecules aggregate or remain dispersed. For example, the predominance of anionic proteins in serum and the cytosol likely helps maintain repulsion and prevents nonspecific aggregation, whereas cationic proteins often function by binding to negatively charged surfaces like the phosphate backbone of DNA or the inner leaflet of the plasma membrane.

#### 2.1.2 Studying Biomolecular Interactions

Studying biomolecular interactions requires techniques that balance sensitivity, accuracy, and practicality. There is a plethora of techniques that can be used. Later we focus on AUC, but here we touch on the advantages and disadvantages for those we commonly use in the Biomolecular Interaction Centre at the University of Canterbury. We characterise the techniques commonly used to understand biomolecular interactions into 1) structural methods, which define the type of interaction through observation of the molecules involved, and 2) binding methods, which define the properties of the interaction (e.g., the affinity of the partners to interact). Usually, multiple methods are used, and what is chosen depends on the questions being asked. Table 1 summarises some of these methods.

**TABLE 1:** Approaches we use at the University of Canterbury | Te Whare Wānanga o Waitaha to study biomolecular interactions.

Technique	Information gained	Sample needs (volume; concentration)	Best uses and advantages	Disadvantages
*Structural methods*				
X‐ray crystallography	Structural details	Hundreds of µL; >10 mg/mL	High resolution structures of stable, soluble complexes; atomic resolution structures of protein‐ligand interactions to drive drug development	Crystal formation and protein diffraction are bottlenecks
Single‐particle electron cryo‐microscopy (cryo‐EM)	Structural details	Tens of µL; >1 mg/mL	High resolution structures of proteins without the need for crystal formation; particularly useful for the structural study of membrane proteins; multiple complexes and conformations can be gleaned from single dataset	Reliance on overseas instruments; expensive; limitations for small proteins
Small‐angle X‐ray scattering (SAXS)	Shape and size information	Tens of µL; >0.5 mg/mL	Identify conformational changes; provide dimensions of biomolecules in solution	Low resolution technique; sensitive to aggregation
*Binding methods*				
Microscale thermophoresis (MST)	Binding affinity (*K*_D_) via thermophoretic shifts.	∼4 µL per point; nM–µM	Quick *K*_D_ screen, small sample volume; no immobilisation	Limited dynamic range; sensitive to capillary fouling; fluorescent labeling needed
Isothermal titration calorimetry (ITC)	Binding affinity (*K*_D_) via heat change, thermodynamics (Δ*H*, Δ*S*), stoichiometry	Hundreds of µL; hundreds of µM	Resolve composition and stoichiometry; Label‐free; full thermodynamic profile	High sample demand; buffer matching is critical; poor for very tight binding (≤pM)
Surface plasmon resonance (SPR)	On/off rates, binding affinity	Tens of µL; tens of µM	Label free; provides *k*_on_, *k*_off_, *K*_D_; relatively low sample requirements	Surface immobilisation required; added complexity for membrane proteins; needs high purity
Bio‐layer interferometry (BLI)	On/off rates, binding affinity	Tens of µL; nM–µM	Label free; provides *k*_on_, *k*_off_, *K*_D_; relatively low sample requirements	Surface immobilisation required; less sensitive than SPR for small molecules.
Size exclusion chromatography coupled with multiangle light scattering/dynamic light scattering (SEC‐MALS/DLS)	Molar mass, stoichiometry, diffusion	∼100 µL; µM range	Confirm molar masses or diffusion coefficients for pure samples; convenient	Resolution limits mixtures; needs high purity
Electrophoretic mobility shift assay (EMSA)	Verify binding; affinity	Tens of µL; nM range	Detect DNA:protein binding quickly; very low reagent requirements; good for comparative or qualitative binding experiments	Limited resolution; no quantitative mass or stoichiometry data
Analytical ultracentrifugation (AUC)	Stoichiometry, molar mass, binding affinity, on/off rates, diffusion, shape	∼0.5 mL per cell; nM–µM	Determining stoichiometry and binding affinity; separates species by size and spectrum (e.g., protein DNA); detects contaminants	Requires accurate extinction coefficients; complex analysis; if fluorescence is used then protein needs to be labelled

The *resolution limit* of an optical system is defined by the smallest distance between two disparate points that the system can distinguish. It is directly proportional to wavelength, as described by Ernst Abbe (Abbe 1873).

Structural methods we commonly use include X‐ray crystallography, cryo‐electron microscopy, and small‐angle X‐ray scattering. The particles of interest (usually proteins) are typically much smaller than the wavelength range of visible light (∼380 to ∼760 nm) (Sliney et al. 2016). This limits the utility of light microscopy as a technique for studying these molecular particles, as the *resolution limit* is intrinsically linked to wavelength. However, the X‐rays and electron beams used by structural biologists have much shorter wavelengths. For example, the wavelength of an electron beam generated by a 300 kV electron microscope is 0.0197 Å (0.00197 nm) (Takaba et al. 2020). These technologies enable scientists to resolve biomolecules to ∼1 Å (0.1 nm), although more typically we see biomolecular structures with resolutions between 2 and 5 Å using X‐ray crystallography and single‐particle cryo‐electron microscopy. Many reviews have been written on these structural techniques, and we point to a few that the reader may find interesting (Shi 2014; Nogales and Heres 2015; Da Vela and Svergun 2020).

*Binding affinity* is the strength of a specific interaction between two (or more) biomolecules. It is represented as *K*_D_, the dissociation constant. For single site binding, *K*_D_ is defined as the following: $K_{D}=\frac{\left[A\right][B]}{\left[AB\right]}$ where [A], [B], and [AB] represent the concentrations of free *A*, free *B*, and complexed *AB*, respectively, at equilibrium (*i.e*. equal association and dissociation rates). Mathematical manipulation also allows us to think of *K*_D_ as the concentration of *A* at which half of *B*'s binding sites are occupied. For example, at 50% occupancy of all *B* sites, the concentration of free *B* equals the concentration of complex *AB*. If [B]=[AB], then substituting into the *K*_D_ equation yields $K_{D}=\frac{\left[A\right][AB]}{\left[AB\right]}$ or simply $K_{D}=[A]$.

Binding methods that serve to characterise biomolecular interactions exploit some type of signal that distinguishes the unbound components from the bound complex. Methods such as microscale thermophoresis (MST) and isothermal titration calorimetry (ITC) are widely used for determining *binding affinities* (*K*_D_). MST measures how molecules move along a temperature gradient. MST is fast, requires minimal sample volumes, and works in solution without immobilisation, making it ideal for quick screening. However, its dynamic range is limited; its accuracy drops for very tight interactions, and it requires that one of the components in the system be fluorescently labelled (Jerabek‐Willemsen et al. 2014; Baaske et al. 2010). ITC works by measuring the temperature changes that occur with binding events. In contrast to MST, ITC provides a full thermodynamic profile—including enthalpy, entropy, and stoichiometry—without labelling, but the technique demands large sample volumes and struggles with extremely strong (i.e., tight, *K*_D_ < 1 nM) binding (Bastos et al. 2023). Complementary methods like size exclusion chromatography coupled with multiangle light scattering/dynamic light scattering (SEC‐MALS/DLS) measure molecules’ movements through a porous medium and in tandem how they scatter light. DLS provides diffusion information, yielding molecular size (hydrodynamic radius), and MALS yields molar mass as well as molecular size (radius of gyration), but the accuracies of these techniques depend on sample purity (Stetefeld et al. 2016; Matson et al. 2024). Surface plasmon resonance (SPR) is a robust technique to measure binding events; it is label‐free and measures the kinetics of biomolecular interactions. SPR requires that one of the components is surface immobilised to a biosensor and exploits changes in refractive index at the biosensor that occur upon a biomolecular interaction to provide binding measurements (Capelli et al. 2023). Bio‐layer interferometry is similarly robust, uses a surface‐immobilised component, but measures the interference of white light reflected from the biosensor tip; as molecules accumulate on the biosensor, the interference pattern shifts (Bates et al. 2025). Electrophoretic mobility shift assays (EMSA) aid in the investigation of protein–nucleic acid interactions. Protein‐nucleic acid complexes typically run differently on a gel than nucleic acids alone (Hellman and fried 2025), and an analysis of the gel shifts of a macromolecular complex at varying protein or DNA concentrations can yield an apparent interaction affinity. EMSAs are discussed in more detail during Case Study #2. AUC, which is the focus of this commentary, analyses particles’ movements as they are subject to centrifugal forces up to 290,000 × *g* and yields information on the size, shape, and interactions of individual macromolecules or complex mixtures of interacting or noninteracting species (Ralston 1993).

Overall, it is key to remember that no single technique captures all aspects of an interaction; combining complementary approaches yields the most robust and reliable characterisation (Demeler 2025).

#### 2.1.3 What is Analytical Ultracentrifugation and Why is it Useful?

AUC operates in different optical systems. *Absorbance* optics measures optical density at various wavelengths as particles migrate during an experiment. *Fluorescence* optics operates similarly but detects fluorescence as opposed to measuring absorbance. *Rayleigh interference* optics measures changes in refractive index that occur as local sample concentration changes during an AUC experiment.

**FIGURE 1:**
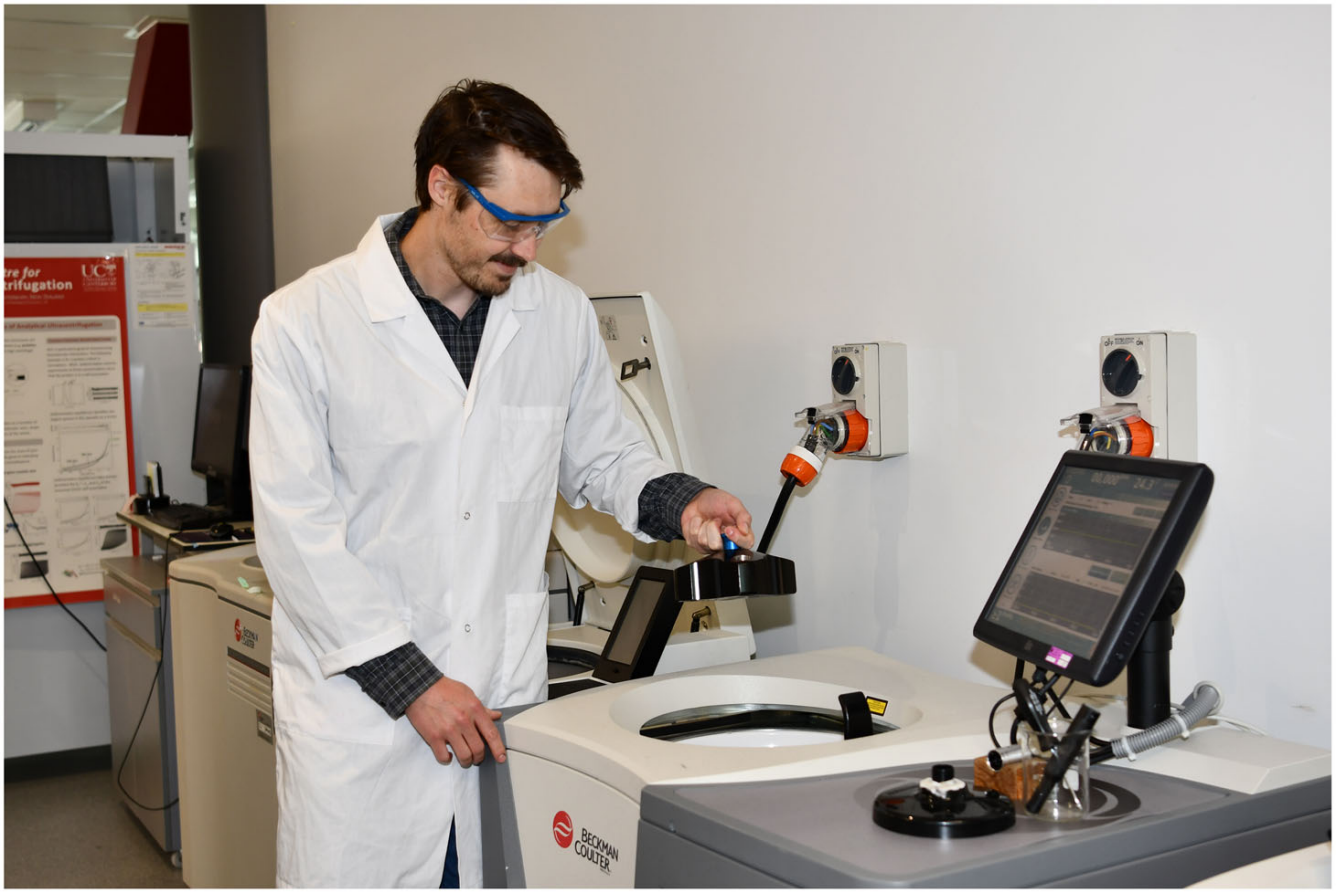
Co‐author, Zachary Tillett, loading a An‐60 Titanium Rotor into the Optima AUC analytical ultracentrifuge housed in the Biomolecular Interaction Centre within the School of Biological Sciences at the University of Canterbury | Te Whare Wānanga o Waitaha.

**FIGURE 2:**
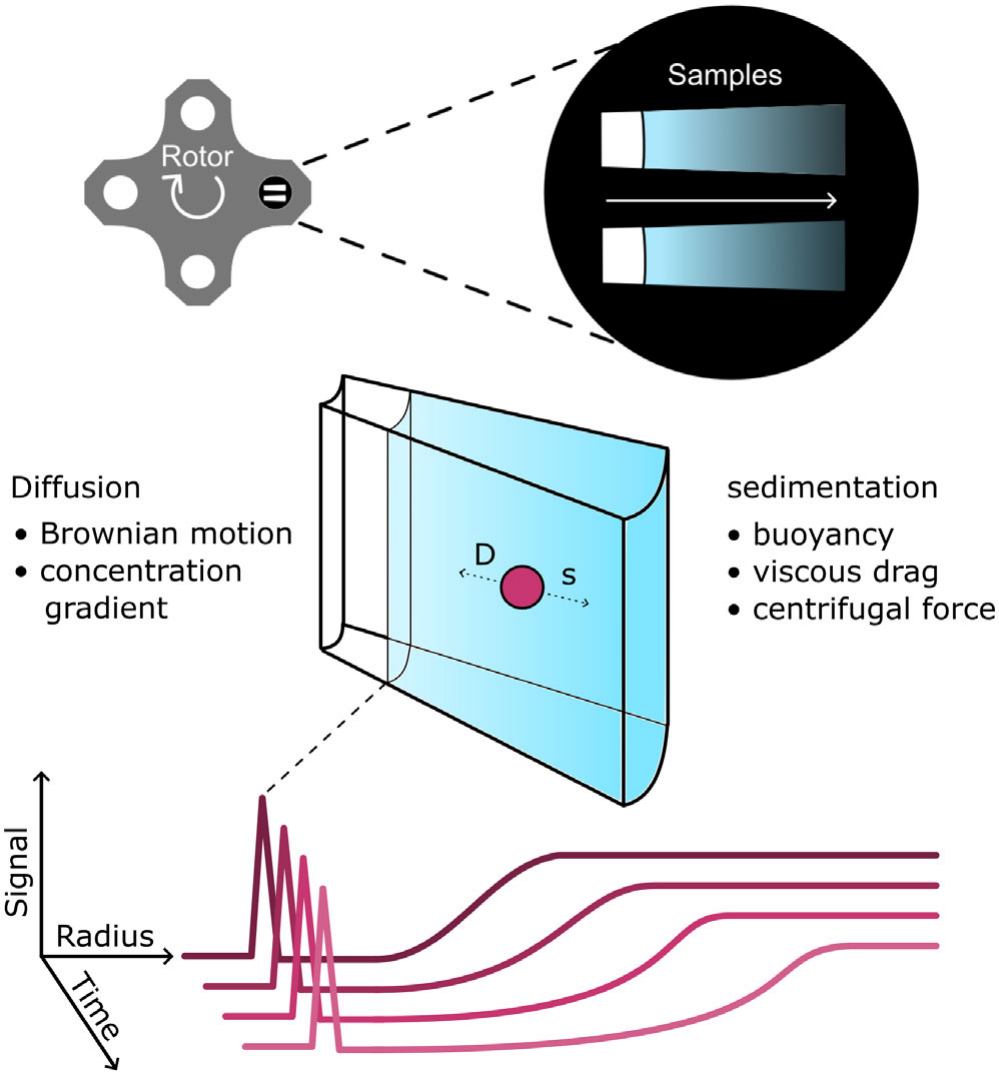
Factors that influence an AUC experiment.

For a deep characterisation of biomolecular interactions, analytical ultracentrifugation (AUC) stands out, in part because of its simplicity and versatility (Table 1). At its heart, AUC observes the movement of a particle through a solution over time under a given centrifugal force. Particles will move differently depending on their mass, their shape, and the properties of the solution they are moving through. The instruments themselves are also simple; an AUC is an ultracentrifuge that has an optical system (*UV/Vis absorbance*, *Rayleigh interference*, or *fluorescence emission*) to monitor the changes in concentration of a particle across the length of a cell over time (Figures 1 and 2). Since their first use in 1920's, the instruments themselves have not conceptually changed. But that doesn’t mean the field hasn’t evolved. The fluorescence optical system offers superb sensitivity and selectivity, enabling reliable measurements down to picomolar concentrations within complex solutions (MacGregor et al. 2004; Zhao et al. 2014), but one of the interacting partners must be labelled with a fluorescent probe that is compatible with the excitation laser (488 nm), which might introduce confounding factors to the experiment. There are many excellent reviews on AUC and its applications (see, for example, (Ralston 1993; Cole et al. 2008), so we will not labour the theoretical detail. However, it is worth looking at the forces that underpin the technique, which are shown in Figure 2.

The *Svedberg equation* solves for the molar mass [*M*] of a particle so long as the sedimentation coefficient (*s*), diffusion coefficient [*D*], and its partial specific volume ($\bar{v}$) are known and determined under the same conditions, such as during an AUC experiment. It is written as follows: $M=\frac{sRT}{D\left(1-\rho \bar{v}\right)}$ .

Particles will experience various forces when in a centrifugal field (Figure 2). As the rotor spins to speeds as high as 60,000 rpm, particles will move or sediment in the direction of the net forces acting upon the object. Sedimentation speed is dependent on the centrifugal force generated from the rotor spinning, the buoyancy of the particle, and the viscous drag caused by the solution it is moving through. Particles also experience diffusion, which is a product of the Brownian, or random, motion of the particles in solution and the concentration gradient that is established once they start moving away from the meniscus due to sedimentation. A particle's sedimentation coefficient (s) is related to its mass and shape (anisotropy), while its diffusion coefficient (D) is related to its shape (Demeler et al. 2014). These are the key parameters we usually want to know and are formularised by the *Svedberg equation*. The movement of the particles through the solution column results in traces that map the concentration of the particles as a function of radius and time (Figure 2, **pink lines**).

The Beckman–Coulter Optima AUC instrument installed at the University of Canterbury features multiwavelength detection as part of its absorbance optics (Pearson et al. 2015). The ability to collect data over multiple wavelengths during the same run introduces an additional spectral dimension, which is ideal for studying biomolecular interactions (e.g., mRNA‐filled LNP vaccines, or protein–DNA interactions) in solution, even in complex mixtures. Multiwavelength detection substantially increases the data density of an experiment and leads to a significant improvement in resolution over single‐wavelength methods (Gorbet 2015). Both case studies presented herein feature multiwavelength analytical ultracentrifugation (MW‐AUC) to deconvolute the signals coming from different macromolecules.

### 2.2 A Brief History of Analytical Ultracentrifugation in Aotearoa New Zealand

Before we focus on some of the work coming out of our lab, we want to acknowledge the foundational contributions of the early pioneers in AUC in Aotearoa New Zealand.

Although the only instruments now available in Aotearoa New Zealand are housed in the Biomolecular Interaction Centre at the University of Canterbury, there was a time when most institutions, particularly hospitals and clinical laboratories, had an analytical ultracentrifuge. Prior to the establishment of the very simple and fast sodium dodecyl‐sulfate polyacrylamide gel electrophoresis method for determining the purity of a protein in solution, AUC was the best technique. Those in the protein chemistry field may have come across proteins with names such as the soybean 15S, 11S, 7S, and 2S proteins, so called because early work on protein extracts from some sources were defined by how their components behaved during ultracentrifugation. In New Zealand, we have a rich history of discoveries using AUC, and the stories below come from discussions with Dr. Mike Boland and Prof. Geoff Jameson (Massey University), and from Allan Craig's history of the first 50 years of DSIR Biotechnology (Craig 1989).

The first analytical ultracentrifuge in Aotearoa was built for John Lyttleton in the 1950s, then working at the Auckland Industrial Development Laboratory (AIDL), which was part of the Department of Scientific and Industrial Research (DSIR). Lyttleton, a passionate biochemist with a strong inclination toward physics, successfully convinced the Dominion Physical Laboratory to construct him an analytical ultracentrifuge, a task largely undertaken by Joe de Stephano. The machine, modelled on European designs but ingeniously assembled using unconventional materials—including naval weaponry components—underwent rigorous testing to meet safety standards. Following the European practice of “testing to destruction”, the rotor was driven beyond its limits until it fragmented within a lead‐lined compartment, proving the device's reliability (and safety). Once installed in the basement, a hastily built concrete partition was added for operator safety. Equipped with this advanced apparatus, Lyttleton achieved groundbreaking work in plant biochemistry, first distinguishing leaf proteins into two fractions and later, during a fellowship at the California Institute of Technology, identifying Fraction I as ribulose diphosphate carboxylase—the key enzyme in the Calvin photosynthetic pathway responsible for carbon dioxide fixation.

In Palmerston North, a new SpinCo Model E analytical ultracentrifuge was installed in the Plant Chemistry Division and was operational by 1967. It was primarily used by John Lyttleton for studying proteins in pasture plants and by George Peterson in early DNA research. Because the instrument was positioned near a drain connected to a neighbouring lab, where solvents, particularly pyridine, were used in paper chromatography, fumes interfered with the ultracentrifuge's UV optics, causing frequent complaints.

*Schlieren optics* was the original method used in AUC experiments and detected the refraction of collimated light at a sedimentation boundary where there exists a concentration gradient.

*Meniscus depletion* is an AUC method that spins the sample to the extent that solute concentration at the meniscus (air‐liquid interface) is zero. It can simplify analysis to know this boundary condition; however, it introduces confounding factors (e.g. complete sedimentation of sample components).

In the early 1970s, a newer Model E was acquired by the NZ Dairy Research Institute and installed at the DSIR. Initially equipped with *Schlieren optics*, the DSIR instrument was later upgraded by Jim Lewis with a laser system (Lewis 1972), enabling interference pattern analysis for more precise concentration measurements. Mike Boland used this instrument to determine molecular weights of leghaemoglobin from lupin using the classic sedimentation‐diffusion method (Boland 1970), actinidin using the *meniscus depletion* method and interference optics (Boland and Hardman 1972) and later alliinase from garlic (Lancaster and Boland 1990).

The Model E analytical ultracentrifuge in Palmerston North was used until at least 1997 (Harvey et al. 1997), with expertise and operational knowledge residing with the aforementioned Jim Lewis, then affiliated with the Physics Department at Massey University. At this point Beckman Instruments had already put to market their more compact and easier to use XL‐A analytical ultracentrifuge. It was not until 2013 that New Zealand acquired a Beckman Coulter ProteomeLab XL‐I, which is like the XL‐A but also features interference optics. The XL‐I is still operational and housed in the Biomolecular Interaction Centre at the University of Canterbury. In 2022, the University of Canterbury further acquired a Beckman Coulter Optima AUC, which is the instrument used in the case studies that follow.

### 2.3 Case Study 1: mRNA‐Filled Lipid Nanoparticles

The first case study concerns a practical formulation problem associated with mRNA‐based vaccines—are the mRNA molecules packaged properly during formulation? To answer the above question, we investigate the following: 1) are the LNPs loaded with RNA, 2) are empty, unloaded LNPs present in the therapeutic formulation, and 3) can we detect any free mRNA?

mRNA‐filled LNPs garnered mainstream recognition during the COVID‐19 pandemic. Operation Warp Speed (Slaoui and Hepburn 2020) oversaw the rapid development of vaccine technology to combat the COVID‐19 virus. While many scientific achievements contributed to this once‐in‐a‐lifetime project that saved lives, including the use of modified bases in the mRNA transcript, which resulted in a Nobel Prize (The Nobel Prize in physiology or medicine 2023), the rate limiting technology was the validation of a safe, efficacious, and scalable delivery vehicle for the vaccine's mRNA payload (Swetha 2023).

*Lipid* *nanoparticles* (LNPs) and *liposomes* are both nano‐scale spheres in which lipids with polar head groups compartmentalize the structure's interior from the environment. *Liposomes* are generally composed of a lipid bilayer that surrounds an aqueous core. *LNPs* are more complex. Ionisable lipids, phospholipids, cholesterols, and PEGylated lipid are LNP components. LNPs are often loaded with cargo such as RNA and serve as therapeutic delivery systems. *Onpattro* is an LNP therapeutic that functions by encapsulating siRNA to decrease levels of the misfolded transthyretin protein (Urits et al. 2020).

mRNA is one of life's fundamental molecules. The central dogma of molecular biology is simplified into the following: DNA is transcribed into RNA, and RNA is translated into protein (Crick 1970). The molecular integrity of mRNA is notably short‐lived, which makes sense from a biological point of view as mRNA functions as a temporary messenger to translate the permanent genetic code of DNA into functional proteins. mRNA stability and degradation are tightly regulated in cells, since this influences protein expression. mRNA technologies have subsequently become a key pillar of modern vaccine development (Shi 2024). However, the aforementioned instability proves problematic in drug and vaccine development. Encapsulating mRNA in LNPs provides a protective barrier around the mRNA and increases both the bioavailability (potential for increased endocytosis) and stability (compartmentalisation away from degrading ribonucleases) of the mRNA (Hou et al. 2021; Tenchov 2021).

*LNPs* and *liposomes* have been used for decades (Tenchov 2021; Sessa and Weissmann 1968; Bangham et al. 1965), and LNPs have been used to deliver therapeutics and are incredibly valuable from medical and public health perspectives (for a helpful review, see Tenchov 2021). For example, the success of *Onpattro* (Patisiran), an siRNA therapeutic delivered in LNPs for treating polyneuropathies, established LNPs as a mainstream platform for RNA therapeutics and mRNA‐based vaccines (Akinc et al. 2019).

**FIGURE 3:**
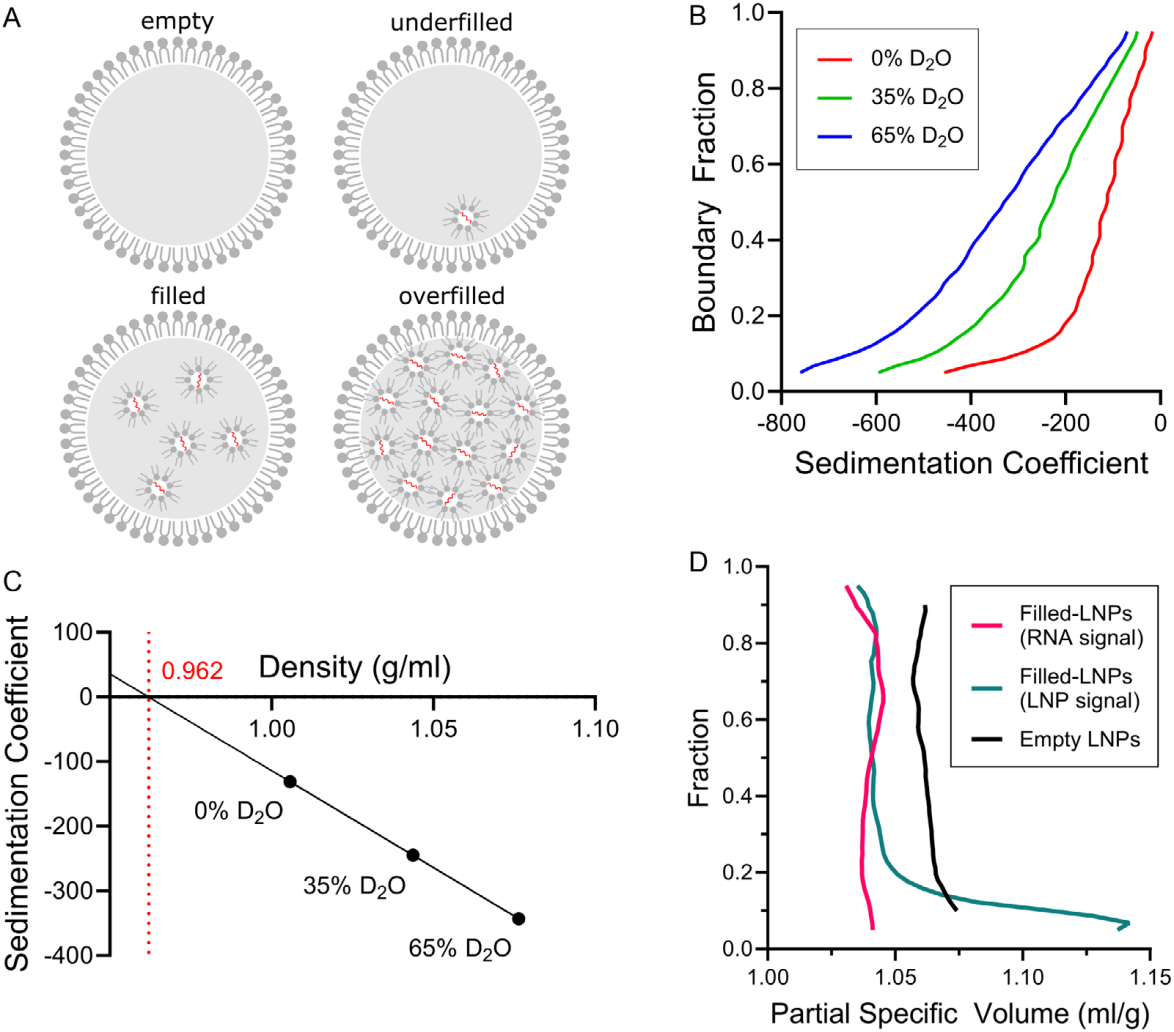
Density matching multiwavelength AUC to validate loading status of LNPs. (A) Simplified schematic of LNPs in various mRNA loaded states. (B) Integral distribution of sedimentation coefficients for mRNA‐filled LNPs at different D_2_O concentrations. (C) mRNA‐filled LNP sedimentation coefficients at increasing D_2_O concentrations are plotted against solution density. Extrapolating to the x‐intercept (0.962, 0) calculates the value at which the sedimentation coefficient is zero, where solution density is equal to mRNA‐filled LNP density. (D) Partial specific volume distributions of filled and empty LNPs are plotted. Multiwavelength AUC was used to deconvolute the data and obtain mRNA‐ or LNP‐specific signal.

The structural integrity of mRNA‐filled LNPs is reliant on biomolecular interactions. Ionisable‐lipids encapsulate the mRNA in the LNP's interior, while phospholipids, cholesterols, and PEGylated lipids create the exterior wall of the LNP. Simply put, phospholipids stabilize the LNP lipid bilayer; cholesterol increases the fluidity of the lipid medium, and PEGylated lipids enhance the stability of the delivery system (Tenchov 2021). For a simplified diagram of the structure see Figure 3A.

At the Biomolecular Interaction Centre, we use AUC to validate the composition of mRNA‐filled LNPs, developed with support from the NZ RNA Development Platform. Preclinical quality control of LNPs requires a rigorous assessment of their physical, chemical, and structural properties (Parot et al. 2024). Particle size and distribution are often used as key quality indicators in the assessment of LNPs, and techniques such as cryogenic transmission electron microscopy (cryo‐TEM) and DLS can be used to determine these indicators. However, clinically relevant, RNA‐loaded LNPs are indistinguishable from their empty LNP counterpart using these techniques, presenting an obvious shortcoming in their analytical rigour. The Food and Drug Administration (FDA) recommends using orthogonal methods to characterise mRNA‐filled LNP formulations (Drug Products, Including Biological Products, that Contain Nanomaterials Guidance for Industry 2022). As previously mentioned, AUC is a high‐resolution technique to measure particle size distribution (Parot et al. 2024), but it also has the capacity to measure LNP density and determine loading status, providing key information where other techniques fall short (Henrickson 2021; Bepperling and Richter 2023). Here, we show how AUC can be used to distinguish mRNA‐filled LNPs from empty LNPs (Figure 3A), as the densities of these particles differ. mRNA‐filled LNPs are denser than empty LNPs and therefore migrate differently during an AUC experiment (Henrickson et al. 2021; Bird et al. 2025), and AUC directly measures these intrinsic, physical characteristics.

Density matching AUC (Brown et al. 2011; Swygert et al. 2014) can be used to experimentally determine whether mRNA‐formulated LNPs are empty or filled (Henrickson et al. 2021; Bird et al. 2025). In a density matching experiment, known quantities of deuterium oxide (D_2_O) are added to the sample buffers, and sedimentation coefficients are determined through sedimentation velocity AUC experiments at the various D_2_O concentrations (i.e., different solution densities, Figure 3B). By plotting solution density on an *x*‐axis and sedimentation coefficient on its *y*‐axis, the *x*‐intercept can be determined by extrapolating the data to a sedimentation coefficient of zero (Figure 3C). When a particle's sedimentation coefficient is zero, it is neutrally buoyant, so its density matches that of the surrounding solution. This calculation of neutrally buoyant density can be done for each corresponding boundary fraction point of the sedimentation coefficient integral distributions (Figure 3B) allowing for dissection of the *partial specific volume* distributions of each sample (Figure 3D) (Henrickson et al. 2021).

*Partial specific volume* or $\bar{v}$ is the increase in solution volume when a unit mass of solute is added and is measured as mL/g. It may be helpful to think of it as the reciprocal of density, and it is what we are solving for in a density matching experiment.

This method accurately determines a particle's partial specific volume (Table 2). Two partial specific volumes are reported for mRNA‐filled LNPs, as these macromolecules were tracked via both mRNA signal contribution and LNP signal contribution using MW‐AUC (Figure 3D). Interestingly, there is a higher partial specific volume when mRNA‐filled LNPs are measured via their LNP signal as opposed to their mRNA signal (Table 2). For both mRNA‐filled and empty LNPs, there is an obvious increase in their partial specific volumes at low fraction values (***y*‐axis,** Figure 3D). This may be explained by how these signals are measured. LNPs do not absorb light like mRNA, but rather they scatter light. The signal from the observed light scattering increases in a nonlinear relationship to particle size, scaling to the sixth power of a particle's radius. This would overemphasize the contribution from larger LNP particles and likely leads to the graph's tails seen at low boundary fractions. Nonetheless, the increase in LNP signal for the mRNA‐loaded LNPs starts at a higher boundary fraction than that of the empty LNP control (Figure 3D). This is likely caused by the presence of ∼20% empty LNPs in the mRNA LNP formulation, which is a key result. The reported partial specific volumes of filled and empty LNPs are very close to each other, but this is expected as the mRNA forms a minor component of the filled LNP (Henrickson et al. 2021; Bepperling and Richter 2023). In a prior study, Bepperling and Richter calculate the composition of an mRNA‐filled LNP to be 95% lipid and 5% mRNA (Bepperling and Richter 2023). Using the same formula, ${\left[\%\right]}_{\left(\text{lipid}\right)}=\frac{{\bar{v}}_{\text{filled LNP}}-{\bar{v}}_{\mathrm{RNA}}}{{\bar{v}}_{\text{empty LNP}}-{\bar{v}}_{\mathrm{RNA}}}$, with our measured values (Table 2), we calculate the mRNA‐filled LNPs used in this study to be 95.5% lipid and 4.5% mRNA.

**TABLE 2:** Calculated densities and partial specific volumes.

Sample	Density, g/mL	Partial specific volume, mL/g
Empty LNPs	0.94	1.07
mRNA‐filled LNPs	0.96 (mRNA), 0.95 (LNP)	1.04 (mRNA), 1.05 (LNP)
Protein	1.41	0.71
Plasmid DNA	2.33	0.43
Free mRNA	2.44	0.41

Free mRNA, empty LNPs, and mRNA‐filled LNPs were provided by the NZ RNA Development Platform. Density values are presented for each analyte following a series of sedimentation velocity experiments at 0%, 35%, and 65% D_2_O. Free nucleic acids have lower partial specific volumes than reported in the literature due to hydrogen deuterium exchange (Henrickson et al. 2021; Largy and Gabelica 2020).

In summary, analysis of LNP size distribution is not trivial and the FDA recommends using orthogonal methods to do so (Drug Products, Including Biological Products, that Contain Nanomaterials Guidance for Industry 2022). At the Biomolecular Interaction Centre, the infrastructure exists to use MW‐AUC to characterise clinically relevant LNPs. Density matching MW‐AUC far exceeds other techniques in the analysis of LNPs because it unambiguously solves for a nanoparticle's partial specific volume in addition to its size; it can therefore differentiate loaded and unloaded LNPs, even if they are similarly sized. This technique can also detect the presence of free mRNA, which was not detected in the mRNA‐filled LNP formulation presented above. While this case study is specific to mRNA‐loaded LNPs, density matching AUC is a robust technique with roots harkening to the 1970s and can be applied to different systems (Brown et al. 2011; Swygert et al. 2014; Edelstein and Schachman 1973; Gohon et al. 2004). We encourage research groups in the region to contact us if they think this technique might prove useful to the questions they are trying to answer.

### 2.4 Case Study 2: Protein–DNA Interactions: Challenges and Opportunities

The next case study showcases how we measure the stoichiometry and affinity of a protein–DNA interaction. DNA encodes genetic information; this information is interpreted through processes that include transcription and translation, and these require proteins to bind to and interact with DNA. There are myriad DNA‐binding proteins with distinct functions, such as nucleases that cleave DNA, histones that package DNA, and polymerases that bind at promoter sequences near the start of a gene and catalyse synthesis of nucleic acid chains. Understanding how proteins interact with DNA, and particularly the stoichiometry of a protein–DNA complex, is key to appreciate the biological role of the interaction (e.g., transcriptional regulation). Here, we present an approach that features AUC with multiwavelength detection to characterise a complex protein–DNA mixture by *deconvoluting the spectral signals* of the two biomolecules (DNA and protein) into separate sedimentation profiles.

*Spectral deconvolution* is the process of separating the absorbance spectrum of a mixed solution into the individual spectra of the mixture's constituent solutes (Henrickson et al. 2022; Mortezazadeh and Demeler 2023).

Regulating gene expression is a fundamental process that enables bacteria to adapt to environmental stresses, such as changes in nutrient availability (Gorke and Stulke 2008; Deutscher 2008; de Lorenzo and Cases 2005). On a molecular level, this process is orchestrated by transcriptional regulators, which are proteins that induce or repress expression of the appropriate metabolic machinery in response to environmental cues, such as the environmental presence or absence of particular sugars (Gerosa 2013; Bervoets and Charlier 2019). To gain a functional understanding of how transcriptional regulators mediate this biological process, validation of the stoichiometry involved in protein‐DNA binding and insight towards the thermodynamics of the interaction is essential for determining the mechanism of action.

To illustrate the potential of this approach, we investigated the interaction between the transcriptional repressor of bacterial sialic acid metabolism, NanR, and its cognate DNA binding sequence. Sialic acids are a family of negatively charged, amino sugars that decorate the surface of mammalian cells, where they mediate a variety of recognition and adhesion processes (Baos et al. 2012; Vimr et al. 2004; North et al. 2018). However, pathogenic and commensal bacteria that colonise sialic acid‐rich environments have evolved elaborate mechanisms to utilise host‐derived sialic acids as a source of nutrition for a competitive advantage (Almagro‐Moreno and Boyd 2009; Vimr et al. 2013). We have been engaged in several studies that examine how sialic acid is metabolised by bacteria, focusing on the mechanism of sialic acid import (North et al. 2018a, 2018b; Wahlgren et al. 2018) and its subsequent enzymatic degradation (Horne et al. 2020; Davies et al. 2019; Coombes et al. 2020; North et al. 2016). Here we review another aspect of our work, where we shifted the focus to examine the mechanism of pathway regulation.

The gene expression of the sialic acid metabolic machinery is regulated by the transcriptional repressor, NanR (Kalivoda et al. 2003; Horne et al. 2021). Our work demonstrated that NanR from *Escherichia coli* achieves gene repression by cooperatively binding a triple‐repeat DNA sequence of GGTATA with high affinity. This interaction involves three NanR dimers in an elaborate, multimeric assembly process (Horne et al. 2021).

**FIGURE 4:**
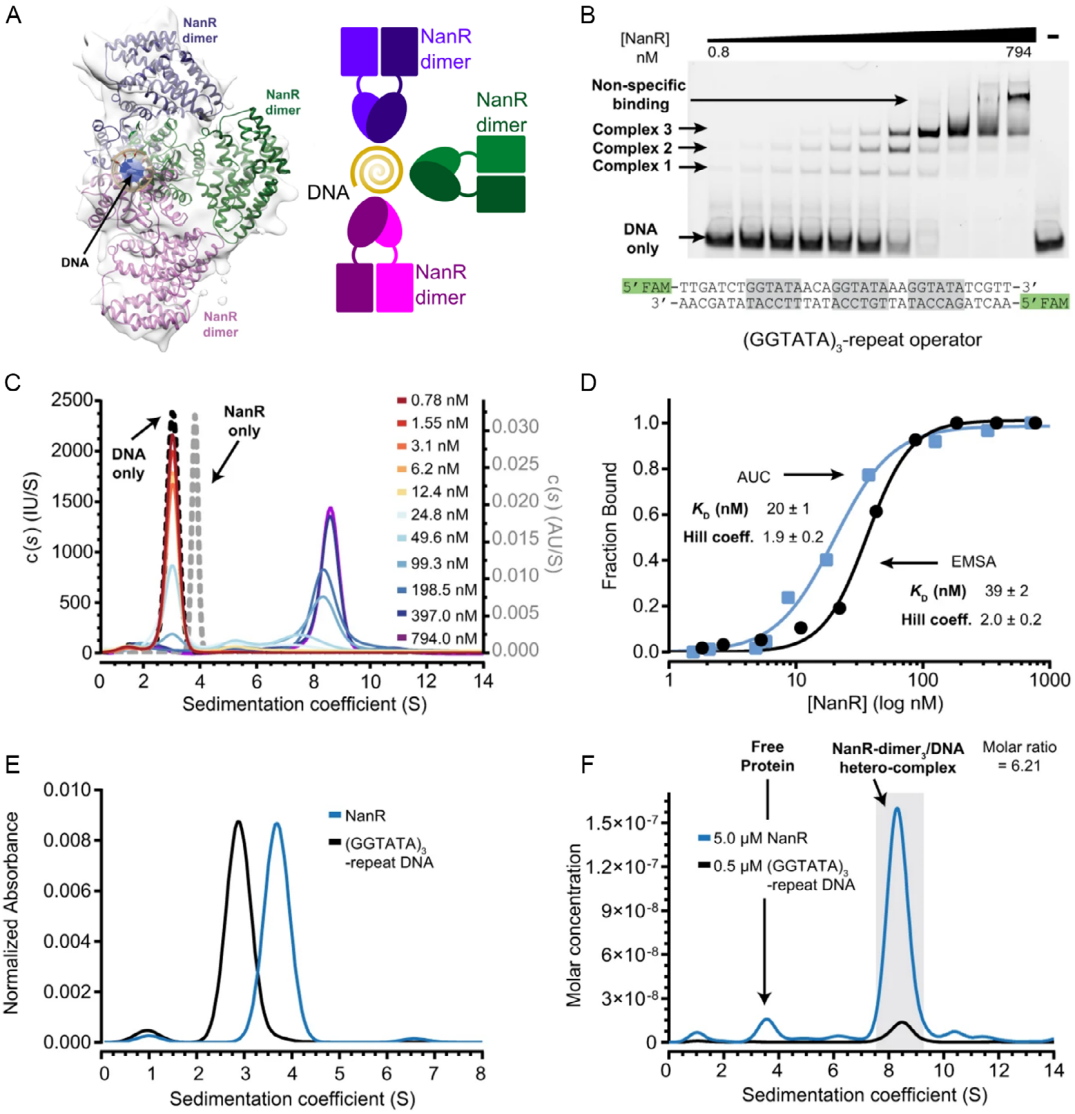
NanR binds DNA as a trimer of dimers (adapted from (Horne et al. 2021). A) 8.3 Å cryo‐EM structure with DNA and three NanR models docked into the density and corresponding schematic. (B) Electrophoretic mobility shift assay (EMSA) shows emergence of multiple protein‐DNA complexes with an increasing concentration of NanR. Nonspecific binding is also observed. 5′ FAM‐labelled DNA sequence with NanR dimer binding sequence shaded in grey is given. (C) Titration AUC experiment shows emergence of higher order species when absorbance at 495 nm is measured to track the 5′ FAM‐label. (D) Binding isotherm data from EMSA (black) and AUC (blue) calculate a nanomolar dissociation constant for the interaction and demonstrate cooperativity (Hill coefficient of 2.0 ± 0.2 and 1.9 ± 0.2, respectively). (E) Control AUC sedimentation coefficient distributions for NanR and DNA alone measured at 280 and 260 nm, respectively. (F) Deconvoluted sedimentation coefficient distributions for NanR and DNA after MW‐AUC of a mixture containing 5.0 µM NanR and 0.5 µM DNA. Integrating the shaded peaks and calculating a 6.21 NanR:DNA molar ratio confirms the stoichiometry of the interaction.

To show that indeed three copies of the NanR dimer interact with its cognate DNA binding sequence we used a combination of structural and biophysical techniques (Figure 4). Low‐resolution (8.3 Å) cryo‐EM density yielded a structural envelope into which we could fit three copies of the NanR dimer bound across the triple‐repeat sequence of GGTATA (Figure 4A). However, the low resolution of the cryo‐EM data limits the confidence of these results, so orthogonal methods are necessary. To confirm the binding mechanism suggested in the cryo‐EM, we conducted an EMSA and AUC (Figure 4B–F). EMSAs, as mentioned earlier, are classic DNA‐binding experiments in which the change in electrophoresis is used as a read‐out for protein binding to DNA. While the method can be powerful in probing DNA–protein interactions and calculating a binding affinity if the experiment is done over a concentration series, it does have limits in accurately measuring the size of the protein–DNA complex formed and determining the stoichiometry of the interaction. Furthermore, EMSAs may fail to detect weak binders and be ill‐suited for quantitative binding studies since they are performed under nonequilibrium conditions. An EMSA clearly showed the formation of distinct complexes in increasing concentrations of NanR and allowed us to calculate a nanomolar affinity (*K*_D_) for the interaction (Figure 4B,D). In fact, the *K*_D_ calculated in the EMSA is within the same magnitude as the *K*_D_ calculated in AUC conducted over a dilution series with absorbance measured at 495 nm to detect the fluorescein (FAM)‐labelled DNA (Figure 4C,D). While in this case, the EMSA provides an accurate binding measure and does suggest stoichiometry, given the trimeric binding pattern with an increasing concentration of NanR, further validation using MW‐AUC was required to confidently assert stoichiometry.

To directly measure the stoichiometry of the protein–DNA interaction, we used MW‐AUC. Protein and DNA controls were measured at 280 and 260 nm, respectively (Figure 4E), and known molar concentrations of NanR dimer and (GGTATA)_3_‐repeat operator DNA were mixed and subjected to MW‐AUC from 220–300 nm. The complex spectra that arise from a MW‐AUC experiment can be deconvoluted using the distinct spectra of the individual species within the mixture (i.e., NanR and the [GGTATA]_3_‐repeat operator DNA). Deconvolution, followed by *two‐dimensional spectrum analysis*, yields molar concentration sedimentation distributions for NanR and its cognate DNA‐binding sequence. Conducted over a series of concentrations (only 5.0 µM NanR:0.5 µM DNA shown in Figure 4F), we can integrate the cosedimenting peaks and calculate a molar ratio. The molar ratio is observed to increase with a factor of two and saturates at six, thus confirming that three NanR dimers are interacting with DNA (Figure 4F).

*Two‐dimensional spectrum analysis* is a model‐independent approach to analyse sedimentation velocity AUC data. This method allows for determination of both a macromolecule's shape (frictional ratio) and its size (sedimentation coefficient) in mono and polydisperse solutions (Brookes et al. 2010)

The example above demonstrates not only the power of AUC, but also the sophistication that experimental planning and the use of orthogonal methods provide to mechanistically detail a complex biological problem. Any of the three techniques used alone would leave unanswered questions and leave us unconvinced of the mechanism of NanR–(GGTATA)_3_ binding. The cryo‐EM suggests a stoichiometry, but it is too low resolution to be assertive. It also does not indicate the strength of the interaction or whether cooperative binding is observed in the system. The EMSA yields an apparent affinity and further supports the stoichiometry modelled in the cryo‐EM. The AUC provides the quantitative rigour that the EMSA lacks, confirming stoichiometry, cooperative binding, and nanomolar affinity in solution.

## 3 Conclusions

The interactions between biomolecules define and determine every aspect of biology, especially at the molecular and cellular levels. As such, studying biomolecular interactions is critical for determining how cells function. Here we have outlined how we tackle complex systems, including mRNA‐loaded LNPs and DNA‐protein complexes. We have focused on our favourite technique, AUC, which is highly versatile and can provide unparalleled information on the biomolecules of interest.

## Funding

The mRNA‐LNP materials prepared for this study were funded from the New Zealand RNA Development Research Platform (Contract RSCHTRUSTVIC2339).

## Conflicts of Interest

The authors declare no conflicts of interest.

## Data Availability

The data that support the findings of this study are available on request from the corresponding author. The data are not publicly available due to privacy or ethical restrictions.
